# Supplementary material for: Global molecular landscape of early MASLD progression in human obesity
Source: eLife. 2026 Mar 23;14:RP109534. doi: 10.7554/eLife.109534 (PMC13008360; doi:10.7554/eLife.109534)
Supplement: Supplementary file 1. [file elife-109534-supp1.docx]

**Table S1**. Additional patient characteristics

| **Characteristic** | **Patients**  **(N)** | **No MASLD**  **(N = 33)** | **MASLD**  **(N = 76)** | **P value** |
| --- | --- | --- | --- | --- |
| ***Additional patient information*** |  |  |  |  |
| Weight (Kg), median ± MAD | 109 | 114.8 (19.57) | 124.35 (26.39) | 0.01 |
| IFG, n (%) | 107 | 0 (0.0) | 5 (6.7) | 0.319 |
| T2DM, n (%) | 109 | 3 (9.1) | 21 (27.6) | 0.058 |
| Cholesteremia history, n (%) | 91 | 2 ( 6.1) | 4 ( 6.9) | 1 |
| ***Metabolic syndrome*** |  |  |  |  |
| MetS triglycerides > 1.7 mM or on treatment, n(%) | 109 | 5 ( 15.2) | 27 (35.5) | 0.055 |
| MetS HDL (HDL < 1.3mM for women or if on treatment), n(%) | 82 | 26 (78.8) | 39 (60.0) | 0.028 |
| MetS HDL (HDL < 1mM men or if on treatment), n(%) | 26 | 4 (12.1) | 22 (28.9) | 0.099 |
| MetS BSL (fasting glucose > 5.6 mmol/L or on treatment), n(%) | 109 | 2 (6.1) | 13 (17.1) | 0.217 |
| MetS BP (hypertension or on treatment), n(%) | 109 |  |  | 0.924 |
| 0 |  | 25 (75.8) | 56 (73.7) |  |
| 1 |  | 7 (21.2) | 17 (22.4) |  |
| 2 |  | 1 (3.0) | 2 (2.6) |  |
| 3 |  | 0 (0.0) | 1 (1.3) |  |
| eGFR (mL/min/1.73m^2^), n (%) | 103 |  |  | 0.656 |
| > 90 |  | 23 (79.3) | 53 (71.6) |  |
| 60 - 90 |  | 5 (17.2) | 19 (25.7) |  |
| < 60 |  | 1 (3.4) | 2 ( 2.7) |  |
| ***Clinical chemistry parameters*** |  |  |  |  |
| HOMA2–%B, median ± MAD | 92 | 101.95 (52.11) | 134.6 (67.38) | 0.342 |
| HOMA2–%S, median ± MAD | 92 | 88.3 (51.97) | 69.75 (46.78) | 0.027 |
| HbA1c (%) , median ± MAD | 91 | 5.2 (0.3) | 5.4 (0.44) | 0.018 |
| Hb (g/L), median ± MAD | 102 | 133 (8.9) | 139 (10.38) | 0.009 |
| Urea (mmol/L), median ± MAD | 104 | 4.3 (1.63) | 4.1 (0.89) | 0.932 |
| Serum creatinine (μmol/L), median ± MAD | 104 | 64 (11.86) | 68 (13.34) | 0.218 |
| Albumin (g/L), median ± MAD | 104 | 38 (4.45) | 39 (4.45) | 0.142 |
| Total bilirubin (μmol/L), median ± MAD | 104 | 6 (2.97) | 9 (2.97) | 0.024 |
| Vitamin B12 (pmol), median ± MAD | 95 | 357.5 (133.43) | 404 (145.29) | 0.12 |
| Vitamin D (nmol/L), median ± MAD | 97 | 59.5 (21.5) | 53 (17.79) | 0.585 |
| Iron (μmol/L), median ± MAD | 102 | 11 (2.97) | 12.5 (3.71) | 0.211 |
| Ferritin (ng/mL), median ± MAD | 102 | 80 (48.18) | 160 (134.18) | 0.004 |
| WCC (*10^9^/L), median ± MAD | 102 | 6.8 (1.78) | 6.8 (2.08) | 0.705 |
| Platelet (*10^9^/L), median ± MAD | 101 | 252 (40.03) | 263 (72.65) | 0.684 |
| MCV (fL), median ± MAD | 102 | 90 (2.97) | 89 (4.45) | 0.449 |
| MCH (pg), median ± MAD | 102 | 29 (1.48) | 29 (1.48) | 0.887 |
| MCHC (g/L), median ± MAD | 102 | 325 (10.38) | 328 (13.34) | 0.188 |
| TSH (mU/mL), median ± MAD | 93 | 1.76 (0.94) | 1.79 (1.07) | 0.121 |
| ***Medication intake records*** |  |  |  |  |
| Angiotensin receptor blockers, n(%) | - | 4 (12.1) | 9 (11.8) | - |
| Calcium channel blockers, n(%) | - | 2 (6.1) | 4 (5.3) | - |
| Other antihypertensive drugs, n(%) | - | 5 (15.2) | 9 (11.8) | - |
| Metformin, n(%) | - | 2 (6.1) | 9 (11.8) | - |
| Statins, n(%) |  |  | 6 (7.9) | - |
| PPI inhibitors, n(%) | - | 6 (18.2) | 10 (13.2) | - |
| Anticoagulants/Antiplatelets, n(%) | - | 3 (9.1) | 3 (3.9) | - |
| Other NSAIDs, n(%) | - | 2 (6.1) | 4 (5.3) | - |
| Ventolin, n(%) | - | 3 (9.1) | 2 (2.6) | - |
| Mental health medications, n(%) | - | 10 (30.3) | 16 (21.1) | - |
| Hormonal medications, n(%) | - | 6 (18.2) | 9 (11.8) | - |
| Duromine, n(%) | - | 1 (3.0) | 3 (3.9) | - |
| Antibiotics, n(%) | - | 1 (3.0) | 2 (2.6) | - |

MetS, Metabolic syndrome; BSL, Blood sugar level; BP, Blood pressure; IFG, Impaired fasting glucose; T2DM, Type 2 diabetes mellitus; eGFR: Estimated glomerular filtration rate; FBG: Fasting plasma glucose; HOMA2 – B: Homeostatic model assessment 2 of beta-cell function; HOMA2 – S: Homeostasis model assessment 2 of insulin sensitivity; WCC, White cell count; MCV, Mean corpuscular volume; MCH, Mean corpuscular hemoglobin; MCHC, Mean corpuscular hemoglobin concentration; TSH, Thyroid-stimulating hormone; PPI, Proton pump inhibitor; NSAIDs, Nonsteroidal anti-inflammatory drugs
